# Factors affecting patients' journey with primary healthcare services during mental health‐related sick leave

**DOI:** 10.1111/hex.14036

**Published:** 2024-04-01

**Authors:** Justine Labourot, Émilie Pinette, Nadia Giguère, Matthew Menear, Cynthia Cameron, Elyse Marois, Brigitte Vachon

**Affiliations:** ^1^ École de réadaptation, Faculté de médecine Université de Montréal Montréal Canada; ^2^ Centre de recherche de l'Institut universitaire en santé mentale de Montréal Montréal Canada; ^3^ Département de médecine de famille et de médecine d'urgence Université de Montréal Montréal Canada; ^4^ Centrede recherche de Montréal sur les inégalités sociales, les discriminations et les pratiques alternatives de citoyenneté. Montréal Canada; ^5^ Département de médecine de famille et de médecine d'urgence Université Laval Québec Canada; ^6^ Vitam—Centre de recherche en santé durable Québec Canada

**Keywords:** mental health, primary healthcare patient journeys, qualitative study, recovery, return to work

## Abstract

**Context:**

Best practice guidelines for the recovery and return to work (RTW) of people with mental disorders recommend access to the services of an interdisciplinary team combining pharmacological, psychological and work rehabilitation interventions. In the Canadian context, primary healthcare services are responsible for providing these services for people with common mental disorders, such as depressive or anxiety disorders. However, not everyone has easy access to these recommended primary healthcare services, and previous studies suggest that multiple personal, practice‐related and organizational factors can influence the patient's journey. Moreover, previous studies documented that family physicians often work in silos and lack the knowledge and time needed to effectively manage by themselves patients' occupational health. Thus, the care and service trajectories of these patients are often suboptimal and can have important consequences on the person's recovery and RTW.

**Objective and Population Studied:**

Our study aimed to gain a better understanding of the patient journeys and the factors influencing their access to and experience with primary healthcare services while they were on sick leave due to a common mental disorder.

**Methods:**

A descriptive qualitative research design was used to understand and describe these factors. Conventional content analysis was used to analyze the verbatim.

**Results:**

Five themes describe the main factors that influenced the patient's journey of the 14 participants of this study: (1) the fragmented interventions provided by family physicians; (2) patients' autonomy in managing their own care; (3) the attitude and case management provided by the insurer, (4) the employer's openness and understanding and (5) the match between the person's needs and their access to psychosocial and rehabilitation services.

**Conclusions:**

Our findings highlight important gaps in the collaborative practices surrounding the management of mental health‐related sick leave, the coordination of primary healthcare services and the access to work rehabilitation services. Occupational therapists and other professionals can support family physicians in managing sick leaves, strengthen interprofessional and intersectoral collaboration and ensure that patients receive needed services in a timelier manner no matter their insurance coverage or financial needs.

**Patients of Public Contribution:**

This study aimed at looking into the perspective of people who have lived or are currently experiencing a sick leave related to a mental health disorder to highlight the factors which they feel hindered their recovery and RTW. Additionally, two patient partners were involved in this study and are now engaged in the dissemination of the research results and the pursuit of our team research programme to improve services delivered to this population.

## 1 INTRODUCTION

In Canada, one person in three will experience a mental illness during their lifetime.^1^ Work disability related to mental health problems is on the rise, with mental disorders now constituting the leading cause of work absenteeism.^2^, ^3^ While the COVID‐19 pandemic may have contributed to this increase,^4^ mental disorders already represented approximately 30% of all sick leave requests in 2013.^5^ Approximately, 25% of mental health‐related sick leaves last longer than 6 months,^6^, ^7^ contributing to long‐term disability and non‐return to work (RTW).^8^

International best practice guidelines for the recovery and RTW of people with mental disorders recommend access to interdisciplinary teams combining pharmacological interventions, psychological interventions, work rehabilitation and concerted actions between partners, such as insurers and employers, involved in the RTW process.^9^, ^10^, ^11^, ^12^, ^13^ In the publicly funded Canadian healthcare system, services for common mental disorders, such as adaptation, depressive or anxiety disorders, are accessed first through primary healthcare services. These services are provided by family medicine groups composed of family physicians and different healthcare professionals^14^ but also include general healthcare, rehabilitation and social services provided in the community and private clinics. When required, family physicians are the main prescribers of sick leave and have a central role in the management of the patient's recovery and RTW trajectory.^13^, ^15^ Following a recovery‐oriented practice model, they should engage in shared decision‐making with patients, ensuring informed access to preferred and appropriate services.^16^ However, in the province of Quebec, access to a regular family physician is limited and over 20% of the population remains unregistered.^17^ Additionally, while family medicine groups offer nursing and social work services, access to psychology and occupational therapy is restricted, often requiring specific private insurance coverage or personal financial resources. Previous studies also documented that family physicians too often work in silos and lack the knowledge to effectively manage patients' RTW process and lack time to help patients navigate the system.^13^, ^15^, ^18^, ^19^, ^20^ In this context, patients' rights to universal quality healthcare are undermined as well as their opportunities for recovery and healthy and safe RTW.^21^ To further understand this situation in the province of Quebec (Canada), our study aimed at gaining a better understanding of the patients' journeys and the factors influencing their access to and use of primary healthcare services while they were on sick leave due to a common mental disorder. Patient journey is defined as the process the patient goes through, or the chain of events the patient experiences, to receive the services needed throughout an episode of care.^22^

## 2 METHODS

A descriptive qualitative research design was used to comprehensively explore patient's perspective of their journey with mental health‐related sick leaves.^23^ Our research team adheres to the recovery model in mental healthcare, which focuses on empowering individuals and supporting them in the resumption of social roles,^24^ and to the work disability paradigm, which explains the complex interactions between personal and environmental factors influencing work‐sick leave and disability.^25^ Master's degree students (J. L., E. P.), involved in the data collection and analysis of this study, were trained in occupational therapy, which potentially influenced their understanding of participants' mental health problems and work disability. However, to mitigate any disciplinary biases on the interpretation, all results underwent rigorous discussion and validation with our interdisciplinary research team composed of two patient partners and researchers from diverse backgrounds (family medicine, anthropology, psychology, occupational therapy, primary care, healthcare services organization).

This research was approved by the ethical committee of the Centre intégré universitaire de santé et de services sociaux de l'Est‐de‐l'île‐de‐Montréal and the Centre intégré universitaire de santé et de services sociaux du Centre‐Sud‐de‐l'île‐de‐Montréal.

The study used purposive sampling and various strategies to recruit patients with diverse insurance coverage and use of services (medical, psychosocial, work rehabilitation, community services). First, a family medicine group and three private rehabilitation clinics were contacted to help identify potential study participants who received different combinations of services during their sick leave. To increase variability in living areas and journeys, advertisement on mental health social network support groups was also used. Patients were eligible if they were 18 years or older, were recently or were still on sick leave for a common mental disorder, were followed by a family physician for their sick leave and spoke French. To ensure that participants could gain some perspective on the services they received, the sick leave needed to have occurred at least 6 months ago, irrespective of whether the individual had already RTW or was still off work.

Two data collection methods were used: (1) a short online questionnaire served to collect information on participants' sociodemographic characteristics and health conditions and (2) a semistructured interview conducted by J. L. and E. P., averaging 1 h in length. The interview guide included questions on the participants' journey and the factors that influenced this journey, their satisfaction and perceived usefulness of the services received and their ideas on how they could be improved. All the interviews were conducted and recorded on the Teams videoconferencing platform.

The initial interviews were transcribed and iteratively coded by J. L. and E. P. Conventional content analysis, as described by Hsieh and Shannon,^26^ was performed through a coding process that facilitated data interpretation, while remaining focussed on the messages conveyed by the subjects. The exact steps followed are depicted in Table 1.

**Table 1:** Steps of the content analysis process.

Steps	Content analysis procedures
Step 1: Read and correct verbatim	Two members of the research team (J. L., E. P.) read the verbatim transcripts to correct errors and reach an agreement on an initial set of codes and a coding framework.
Step 2: Independent inductive coding	Each verbatim transcript was coded independently, using the NVivo software (Version 13),^27^ by one of the two researchers and reviewed by the other for intercoder consistency.^28^ The two researchers consulted each other repeatedly to harmonize interpretations of the results.
Step 3: Validation of codes	The initial set of codes and sub‐codes were reformulated and reorganized during the coding process. Two experienced qualitative researchers on the team (N. G., B. V.) listened to half of the recordings to validate the coding process and ensure that the analyses accurately reported the participants' perspectives.
Step 4: Emergence of themes	The NVivo codes and quotations were then transferred to Word files and the codes were regrouped under preliminary themes in line with our research objectives.
Step 5: Validation of themes	The preliminary findings and interpretations from the content analysis were shared and discussed with the broader team, leading to a final revision of the themes and selection of supporting quotations.

## 3 RESULTS

### 3.1 Characteristics of participants and their access to primary healthcare services

A total of 14 patients who received or were receiving primary healthcare services during their sick leave were interviewed (Table 2). Apart from one man and one nonbinary person, all the participants were women.

**Table 2:** Sociodemographic and sick leave characteristics of each participant.

Participant number	Sociodemographic characteristics				Sick leave characteristics				Professionals seen in sick leave								
	Gender	Age (years old)	Has mental health disorder history	Work title	Diagnosis associated with sick leave	Phase of RTW^a^	Length of SL^b^	Insurance coverage	Family doctor	Psychologist	Psychotherapist	Social worker	Other therapist/groups	Occupational therapist	Kinesiologist	Guidance counsellor	Psychiatrist
1	Female	40	✓	Healthcare manager	Adjustment disorder	Phase 2	>1 year	Private insurance and EFAP	✓	✓	✓			✓	✓		
2	Female	57	✓	Political advisor	Burnout and depression	Phase 2	>1 year	Private insurance	✓		✓		✓	✓		✓	
3	Female	36	✓	Government agent	Generalized anxiety disorder	Phase 2	>1 year	Private insurance	✓					✓			✓
4	Male	49		Director of operations	Depression	Phase 4	>1 year	Group insurance	✓			✓		✓			✓
5	Female	48		Cultural worker	PTSD	Phase 4	>1 year	Public insurance and CNESST	✓	✓		✓	✓	✓			
6	Non‐binary/fluid	29	✓	Psychosocial counsellor	Depression and anxiety	Phase 4	<3 months	No insurance	✓			✓					
7	Female	31		Occupational therapist	Characterized depressive disorder	Phase 3	6 months to a year	Private insurance	✓	✓		✓	✓				✓
8	Female	39		Social worker	PTSD	Phase 2	6 months to a year	EFAP, public Insurance and CNESST	✓	✓		✓	✓				
9	Female	25	✓	Freelance graphic designer	Generalized anxiety disorder, burnout and depression	Phase 4	3–6 months	No insurance (government Employment Insurance benefits and leave) and EFAP	✓			✓	✓				✓
10	Female	28		Medical secretary	Major depression and adjustment disorder	Phase 3	>1 year	EFAP and private insurance	✓	✓		✓				✓	
11	Female	28		Office agent	*None*	Phase 4	<3 months	No insurance and EFAP	✓	✓		✓					
12	Female	41		Advertising representative	Generalized anxiety	Phase 4	<3 months	EFAP and private insurance	✓	✓							
13	Female	36	✓	Psychosocial worker	Generalized anxiety disorder and PTSD	Phase 2	>1 year	EFAP, private insurance and government Employment Insurance benefits and leave	✓	✓		✓	✓				
14	Female	47		Administrative assistant	Major depression	Phase 4	>1 year	Private insurance	✓	✓				✓			

Abbreviations: CNESST, Commission des normes, de l'équité, de la santé et de la sécurité du travail; EFAP, Employee and Family Assistance Program; PTSD, posttraumatic stress disorder; RTW, return to work;

^a^ The phases of RTW are based on the ones described in Corbière et al.^29^: (1) beginning of sickness absence and involvement of disability management team (Phase 1); (2) involvement in treatment and rehabilitation with health professionals, and preparation for RTW (Phase 2); (3) gradual RTW and follow‐up (Phase 3). Phase 4 was added to this study to describe people whose RTW and rehabilitation process is now complete, either because they have RTW or have quit their previous work.

^b^ This represents the length of the sick leave at the time of the interview, as sick leaves could still be ongoing.

As seen in Table 2, participants were diverse with respect to their work situations before their sick leave and insurance coverage. Twelve participants had a family physician managing their sick leave, but only seven were able to obtain an appointment with them to prescribe their sick leave. Nearly all the participants (*n* = 13) received a diagnosis related to their sick leave, most of them being an adjustment disorder or depression. At the time of the interviews, only one participant had returned full‐time to the same job. Six participants had terminated their employment relationship, since they decided to change workplace or type of employment. Two were in the process of a gradual RTW and/or still had follow‐ups with professionals. The other five were still on sick leave.

Most of the participants received some kind of psychosocial services (psychologist [*n* = 10], psychotherapist [*n* = 2], social worker [*n* = 9] and other [*n* = 5]), and a few received some kind of work or physical rehabilitation services (occupational therapy [*n* = 6], kinesiology [*n* = 1], guidance counselling [*n* = 2]). Nine reported they had referred themselves to these services, five were referred by the insurer and only four were referred by their family physician. As their initial services during their sick leave, seven participants self‐referred to their Employee and Family Assistance Programs (EFAP), which is a confidential, short‐term, counselling services programme paid by employers. Four participants also had a consultation with a psychiatrist.

### 3.2 Factors influencing participants' journeys

Our analysis revealed five themes describing the main factors influencing participants' journey: (1) the fragmented interventions provided by family physicians; (2) patients' autonomy in managing their own journey; (3) the attitude and case management provided by the insurer; (4) the employer's openness and understanding and (5) the match between the person's needs and their ability to access to psychosocial and rehabilitation services. The themes and their subthemes are presented in Table 3.

#### 3.2.1 Fragmented interventions provided by family physicians

The first factor identified as influencing their journey is the practices of the family physician, as the physician was responsible for prescribing and renewing the sick leave. Three subthemes were identified as reported in Table 3.

*Timely access to the family physician*: Participants first identified difficulty in having timely access to their family or attending physician when their mental health status interfered with their work. This led two participants to resort to alternatives such as to continue working, taking vacation days or unpaid leave. This increased their distress by adding a sense of powerlessness and frustration.

When I called there, […] it was hard to make any phone calls. […] But I managed to call, and then I almost had to get mad to obtain an emergency appointment at the clinic […] I couldn't believe the healthcare system was unable to fit me in within three days to see a doctor. (Participant 8)

*Being listened to and treated with compassion in the decision‐making process*: The participants (*n* = 11) stated that physician often questioned them about their perceived ability to work to prescribe or renew their sick leave. This method made participants feel listened to in the decision‐making process. Five participants stressed the importance of being treated with compassion and absence of judgement by their physician as helpful factors in their recovery process.

She listens to me. She doesn't judge me. You know, that's something I find important. After all, you're talking about something that's pretty personal. You know, being judged isn't much fun. It does not make you want to talk about it. (Participant 11)

*Physicians' limited capacity to ensure access to services and collaborate with the other stakeholders*: Only four participants reported having been referred to other professionals by their physician. These were made right at the beginning of the medical follow‐up and gave them a greater sense of being supported and assisted by their physician. One participant (Participant 4) explained his physician's quick referral to a social worker by the fact that they worked together in the same family medicine group. Six participants reported that faster follow‐up could have prevented or at least facilitated their recovery process.

[I had] absolutely no services, so it was all very complicated. I had begun to have some pretty dark thoughts because, you know, I had no resources. (Participant 10)

According to some participants (*n* = 6), physicians' lack of referrals to other professionals could stem from their lack of time and knowledge of existing and accessible services. Indeed, three participants pointed out differences noticed between physical and mental health‐related sick leave experiences.

[When] I had cancer, […] it was my family doctor who did the liaising between all those wonderful professionals. Because it was physical. But when it's [a question of] mental health, the doctors, they… hmm… they're just so‐so. (Participant 1)

Two participants also criticized their physician's lack of competence and referrals to more specialized professionals (e.g., psychiatrists or pharmacists) to adjust medication when needed.

Trying the medication for seven months and it didn't work, when I could have been referred right away to a psychiatrist, who could've solved my problem directly, and I would've been able to take advantage of my [sick] leave to rest. (Participant 9)

However, it is noteworthy that some participants viewed their physician's lack of referrals to other professionals as justifiable when they believed they had the personal or environmental resources to seek services independently.

#### 3.2.2 The person's autonomy in managing their own journey

Participants described how they played a key role in orienting their own services and how their capacity to mobilize their resources, as well as their level of comfort in navigating the health and social services system, influenced the quality of services they received. This was reported to be especially difficult at the beginning of their sick leave because of their mental health status. Two main subthemes were identified in relation to their capacity to manage their journey as reported in Table 3.

*Capacity to mobilize their personal, social and financial resources*: First, participants with experiential knowledge, whether from working in the health and social services system (*n* = 6) or from previous episodes of mental illness (*n* = 6), felt better equipped to identify and refer themselves to services that would address their needs. For example, two participants already had knowledge allowing them to plan their own gradual RTW. They however expressed that they would have preferred to receive support from a professional. People with previous experience of mental disorder reported understanding approaches that worked best for them and returning to professionals who understood their needs.

I've done a lot of therapies in my life. I've done psychoanalysis, I know it by heart […] That really wasn't the right person for me. That's why I'd prefer someone who's working more on the practical level than on the analytical level. (Participant 2)

Moreover, support from a loved one, especially when they worked or were familiar with the health and social services system, also helped the person on sick leave identify and access different resources. Several participants (*n* = 4) expressed having requested a sick leave or consulted a professional considering recommendations made by their loved ones.

Financial resources were also reported to facilitate access to certain services and allowed the participant to choose services adapted to their needs without constraints imposed by insurance. Those with fewer financial resources (*n* = 3) were obliged to use services chosen by the insurer, or, for those with no insurance, to choose services in line with their ability to pay (e.g., seeing a relationship therapist instead of a psychologist).

*Ease in navigating the system*: Finally, the person's proactivity influenced their access to and the quality of the services received in addition to facilitating their own recovery. Notably, the participants who went to their physicians prepared, had some previous medical knowledge and were able to clearly express their needs had better experiences with their physicians.

Every day, I made notes, a table of how I felt […]. I gave [the doctor] that; she thought it was wonderful because [it meant that] the appointment wasn't too long; she read it, then afterwards we came up with a diagnosis. (Participant 3)

#### 3.2.3 The attitude and case management provided by the insurer

Participants also mentioned that their insurer had a major influence on their journey. Two subthemes were identified as seen in Table 3.

*The influence of a rehabilitation counsellor*: Two of the five participants, who had access to a rehabilitation programme through their employer, were referred within a month by the insurance agent to a psychologist or psychotherapist. More often, rehabilitation counsellors working for the insurer were only available after 6 months of sick leave, thus delaying access to services such as occupational therapy. The rehabilitation counsellors also helped them identify their needs and redirect them to the appropriate professionals.

At the moment when I said that I wasn't really making any more progress with the social worker, that I found I was sort of at a standstill […] the insurer proposed the occupational therapist. (Participant 4)

*The influence of insurance coverage*: The availability and type of insurance coverage had a considerable impact on patients' journey. Participants whose insurance plan offered neither a rehabilitation counsellor nor a work rehabilitation programme (*n* = 2) had to find the types of services they needed on their own and had insufficient funds to allow a reasonable length of follow‐up with, for example, a psychologist.

I didn't have […] the care that I could [have had], and, you know, the psychologist […] charged $150, but [the insurance coverage] was used up after three or four sessions. (Participant 13)

Three participants, who had no insurance coverage, had limited access to primary healthcare services due to difficulties in identifying their needs and lack of funds to cover professionals' fees. Their salary was compensated under government programmes, such as health insurance and unemployment insurance, for a maximum duration of 3 months. One of them had to shorten their sick leave despite their health condition (Participant 9), while another had to go on social assistance (Participant 6).

*The timeframes set and undue pressure applied by the insurer*: Four participants reported having to wait an average of 3 months for their cases to be approved for compensation. This put them in a financially precarious position, which induced stress and a feeling that the validity of their condition was called into question, two elements that hindered their recovery. Five participants experienced pressure from insurers to quickly RTW, causing significant recovery issues due to frequent calls, pressure and a sense of untrustworthiness.

[The insurance agent] was super nice and understanding on the phone. But you know, I really felt when I said, ‘Well, yes, I'm managing well with my kids. Yes, I'm able to go for a walk. Yes, I'm able to continue my studies’ [that I had to] return to work then. (Participant 13)

#### 3.2.4 The employer's openness and understanding

The employer's perceived understanding of the issues associated with mental health‐related sick leave, as well as its attitude towards RTW, was seen as influencing participants' journey. Two subthemes were identified as seen in Table 3.

*The employer–employee relationship influencing the person's RTW goals*: Five participants reported having conflictual relationships with their employer before their sick leave, which impacted their RTW goal and self‐efficacy. They believe they could not go back to the same work environment and that maintaining their employment relationship was detrimental to their recovery. However, this employment relationship was sometimes essential to access psychosocial and rehabilitation services.

The occupational therapist knew it, the social worker knew it, the doctor knew it, that I was dragging a ball and chain, and what was this ball and chain? It was the employer. And [that] as long as this ball and chain was still attached to my leg, I'd probably have a hard time continuing my journey. (Participant 4)

*The need for the employer's support to identify and manage gradual RTW arrangements*: Three participants received support from their manager during progressive RTW in the form of understanding comments and attentive listening, which made them feel respected in their recovery process. However, two participants explained that their manager was ill‐prepared or lacked resources to effectively support them in their RTW.

The RTW program, it's like ‘Now, you're gonna work two days, but you gotta work [hard]. I give you back all your accesses, so now let's go! It's business as usual’. So I get back all my clients, everyone reach out to me at the same time, but I only work two days […]. I mean come on it was ridiculous. (Participant 12)

One of the participants (Participant 14) reported that their manager was reassured by the occupational therapist's intervention and insisted on her collaboration during progressive RTW.

#### 3.2.5 The match between the person's needs and their ability to access psychosocial and rehabilitation services

**Table 3:** Presentation of themes and their subthemes.

Themes	Subthemes	
1. Fragmented interventions provided by family physicians	1.1	Timely access to the family physician
	1.2	Being listened to and treated with compassion during the decision‐making process
	1.3	Physicians' limited capacity to ensure access to services and collaborate with the other stakeholders
2. Patients' autonomy in managing their own journey	2.1	Capacity to mobilize their personal, social and financial resources
	2.2	Ease in navigating the system
3. The attitude and case management provided by the insurer	3.1	The influence of a rehabilitation counsellor
	3.2	The influence of insurance coverage
	3.3	The timeframes set and undue pressure applied by the insurer
4. The employer's openness and understanding	4.1	The employer–employee relationship influencing the person's RTW goals
	4.2	The need for the employer's support to identify and manage gradual RTW arrangements
5. The match between the person's needs and their ability to access psychosocial and rehabilitation services	5.1	The importance of multiple types of professional expertise
	5.2	The varying psychosocial support approaches used
	5.3	The occupational therapist's intervention as a support in resuming activities and coordinating services

Finally, the types of professionals seen and the timing of their interventions influenced patients' journeys and their attainment of a level of recovery conducive to an RTW. Three subthemes were identified in this regard as seen in Table 3.

*The importance of multiple types of professional expertise*: All participants stressed the importance of receiving multiple types of services along their journey to maximize recovery and support during the sick leave. They acknowledged, however, that they had difficulty identifying their needs, limited knowledge about the roles of different professionals and would, therefore, benefit from having support in identifying the right approach to help them in their recovery and RTW process.

Being able to name our priorities, it's not easy and name our needs and recognize them […] not everyone has this ability. […] There are plenty of psychologist, plenty of therapies, plenty of professionals. [We need] a resource person who can really help, a bit like a guidance counsellor, but for health. (Participant 7)

Three participants reported that exposure to new professionals was especially beneficial when experiencing stagnation or difficulties in stopping a follow‐up with professionals who no longer provided effective support for their recovery. They reported needing support to access other services since they often experienced lack of energy to find another therapist, lack of awareness of other approaches or professionals that could help, and difficulties in identifying that they had reached a plateau.

You're not well enough, and you're in no state to decide. Basically, you're not in a state to decide for yourself if this psychologist is right for you. (Participant 5)

*The varying psychosocial support approaches used*: The participants' experience with psychosocial professionals, including psychologists, psychotherapists, social workers and relationship therapists, was generally positive as it helped them become aware of certain behaviours or thought patterns that they had. This awareness, in turn, facilitated their decision‐making process and ability to set limits, two elements perceived as important for recovery and RTW.

I knew that when I went back [to work] my symptoms would return. So for me, it was like a, a no‐go; that's kind of what I talked about with the psychologist all along. (Participant 12)

The five participants who identified receiving a ‘concrete approach’ (i.e., interventions focusing on practical solutions) were satisfied with their experience, whereas three, out of the five participants who did not see any impact of psychosocial interventions, felt they were simply being listened to.

It wasn't concrete enough for me [the psychologist's approach]. He was a good listener, maybe for someone who just needs to let off steam and to be heard, well then it works. [But] I needed concrete tools to get better. (Participant 1)

Furthermore, participants who used the EFAP services from their employer (*n* = 7) often reported low level of satisfaction due to limited sessions and inappropriate approaches to help them solve the problems they faced during their sick leave.

When you do short‐term follow‐up, you can't start a psychoanalysis, you know. It's like [if] you open up a Pandora's box and then you leave it open. (Participant 1)

It should be noted that the participants' opinions on each of the professionals varied greatly, depending on their prior experiences and personal preferences. However, participants' discourse revealed the importance of the therapist adopting an approach adapted to the person's needs and establishing a good therapeutic relationship with the professional.

You know, that's the type of approach I needed. You know, we all have our favorite approaches, sort of, but in my case, it was hers. It really suited me a lot. (Participant 13, talking about his social worker's approach)

*The occupational therapist's intervention as a support in resuming activities and coordinating services*: Concrete interventions aimed at developing their functional capacities and preparing them for a possible RTW were only offered to the participants referred to an occupational therapist (*n* = 6). This type of intervention was identified as key to their recovery.

My occupational therapist helped me find ways to organize myself in day‐to‐day life so that I could be functional. She found ways to help me manage my energy (…) and ways to reactivate my cognitive abilities. (Participant 1)

They also reported that their occupational therapist helped them to manage their sick leave by facilitating their navigation of services (*n* = 6), contacting the workplace and negotiating with their insurance (*n* = 2), partially relieving them of this responsibility.

The fact that she's the one who took all those steps, well, that was super helpful, because those were some of the things I still had trouble doing, mobilizing myself to take steps, you know. (Participant 1)

Four of them knew nothing about the role of the occupational therapist until they were referred to one, but based on their experience, they would recommend early referral to these services due to their holistic view of the person's functioning, including work capacity.

(…) the occupational therapist, I found [experience] amazing because she was the first person who took [my housing, my primary needs] into account. And that's how I was able to move forward. (Participant 5)

## 4 DISCUSSION

This study sought to explore patients' perspectives of their journey with primary healthcare services in the context of a sick leave related to a common mental disorder. Our results revealed that patients experienced a variety of journeys and that they were impacted by several factors, including the engagement of their attending physician, their capacity to mobilize their resources, the presence and management approach of the insurer, the support from the employer and patients' opportunities to access psychosocial and work rehabilitation services. To our knowledge, this is the first time patients' perceptions of mental health‐related sick leave have been studied through a journey lens that explored their experiences and interactions with different professionals and stakeholders over time.

First, our findings support the view that family physicians play a central role in the management of common mental disorders and in sick leave‐related trajectories.^30^ Ensuring that people experiencing work‐related stress or burnout can consult a family physician and receive timely, compassionate care from that physician was seen as important to promote wellness and recovery. However, patients also reported that family physicians' coordination and collaborative practices related to sick leave were limited. This lack of coordination often placed the burden on the patient themselves, which was less than ideal for most participants given their vulnerable mental state and their expressed need for support in identifying their own needs and coordinating care. Participants felt that family physicians may lack the skills or training to effectively manage and coordinate mental health and work‐related issues, a finding that is consistent with previous studies.^13^, ^31^, ^32^ For example, this has been raised in a recent Swedish study, aimed at documenting organizational factors influencing return‐to‐work coordination processes, which found that lack of collaboration, long waiting times and inadequate drawing of RTW plans by primary care physicians were perceived as main barriers to better RTW coordination.^33^ Other factors that may explain these suboptimal practices include the lack of time available during consultations and physicians' fee‐for‐service remuneration models, which can limit physicians' level of engagement in the management of their patients' mental health‐related sick leave.^13^, ^15^, ^34^

Second, we found that access to mental health‐related sick leave services varied in function of the level of involvement of different stakeholders and professionals. As mentioned by Corbière et al.,^30^ even if all stakeholders share the interest of achieving safe and sustainable return to work, the collaboration between them is difficult because each stakeholders' role are not well defined and understood by everyone. Furthermore, our results showed that these providers often missed opportunities to intervene at the opportune moment to truly meet patients' needs and support their recovery process. For most participants, their journeys were characterized by significant delays in accessing services, confusion about the roles of the different providers involved, and care and service approaches that were not tailored to their needs. Our results therefore highlight the important variation of services depending on the person's resources and understanding and knowledge of the health and social services system. Indeed, patients with past personal or professional experiences and environmental support were better at navigating care services and had more opportunities. In sum, the participants painted a clear portrait of a fragmented system in which stakeholders involved did not work collaboratively and with a common purpose to facilitate their eventual RTW and promote overall recovery from mental illness.

Third, participants emphasized the roles that insurers had on their journeys by providing support from a rehabilitation counsellor and covering professional fees, even if it was considered as being provided too late. Nevertheless, as documented in the literature, this support was also perceived as undue pressure imposed by insurance companies. This role of the insurer shows the power dynamic that can exist between the patient, the health professionals and the insurer and supports the evidence reporting the importance of communication efforts between all stakeholders.^30^ Additionally, our findings raise doubts about the required need for the employer to maintain contact with the employee while on sick leave to reinforce a feeling of belonging to the organization,^30^, ^35^ as some participants in our study expressed how this was not helpful for their recovery. Therefore, it is crucial to ensure that both insurers' representatives and employers receive improved training on how to provide a supportive, flexible and recovery‐oriented approach rather than a coercive stance focused solely on return to work.^36^

Finally, in line with recommendation from the World Health Organization,^37^ our findings highlight the importance of having a specific actor responsible for coordinating medical, psychosocial and work rehabilitation primary healthcare services. This actor should adopt a recovery‐oriented model approach and be independent of both insurer and employer to facilitate interprofessional and intersectoral collaboration, assess the environmental obstacles to the RTW, and identify possible accommodations or adaptations in collaboration with the employer.^38^, ^39^, ^40^ This case manager role has been previously described in the literature as the ‘return‐to‐work coordinator’ (RTWCo). However, up until now in Quebec (Canada), these RTWCo have generally only been integrated within large organizations to manage the sick leaves of their own employees,^40^ raising once again accessibility and equity issues. Since RTWCo interventions appear to be essential right from the start of sick leave, our results suggest that family physicians and patients would benefit from working in close collaboration with this provider as a member of the primary healthcare team to reduce the management and administrative burden of sick leave. Occupational therapists were found, by participants who benefitted from their services, to take a natural role of coordinating services. Indeed, occupational therapists are trained to evaluate the person's work capacities, analyze work requirements and environment, develop a gradual RTW plan based on the person's work capacities and promote concerted action between stakeholders.^41^ Besides, recent studies revealed that occupational therapists are regarded as the profession with the highest level of competence for this coordination role, and different studies conducted in Canada and the United States also recommend the integration of occupational therapists in family medicine groups.^41^, ^42^, ^43^, ^44^ Having access to the services of an RTWCo would also help reduce inequities in access to primary healthcare services by supporting all patients with different levels of capacity to mobilize their own personal, social and financial resources and ease to navigate the system.

## 5 STRENGTHS AND LIMITATIONS

One strength of our study is the heterogeneity of our sample, in terms of insurance coverage and services used, which allowed us to document various factors influencing the patients' journey. The credibility, dependability and confirmability of the results were ensured by transcribing all the interviews, having several members of the research team listen to the interviews, having two team members iteratively review the coding process, engaging two other members of the team in periodical revision of the coding and validating the results with the entire research team including two patient partners. The thorough description provided of the participants' characteristics and of the study context and results supports the transferability of the results to other contexts with a similar healthcare and social services system.

However, our study had certain limitations. First, while we were able to recruit a sample of participants with different service trajectories, our sample was mostly composed of White women, living in an urban area and registered to a family physician, which can limit the transferability of the results to other populations. Further studies may be required to document other gender and ethnicity perspectives. We also recruited multiple participants working within the healthcare system. This can maybe be explained by the recent COVID‐19 pandemic, which increased the prevalence of mental disorders among healthcare workers. Even with our efforts to recruit participants with more diverse characteristics, we were limited by the profile of the persons who showed interest in participating in the study.

## 6 CONCLUSION

This study describes how people who have been on a mental health‐related sick leave experience and perceive their current care and service journeys. Specifically, our findings first highlight important gaps in the collaborative practices surrounding the management of mental health‐related sick leave. These results also reveal the importance of strengthening coordination of these services, such as through the integration of RTWCos in primary healthcare. Occupational therapists or other professionals in this role could support the family physician in managing the sick leave, strengthen interprofessional and intersectoral collaboration and ensure that patients receive needed services in a timelier manner no matter their insurance coverage or financial needs. Such an approach should be informed by recovery‐based models that are strengths‐based and focused on empowering patients to play an active role in their care and recovery while being supported by accessing timely and appropriate health services.

## AUTHOR CONTRIBUTIONS

**Justine Labourot**: Conceptualization; project administration; writing—original draft; methodology; data curation; writing—review and editing; formal analysis; investigation. **Émilie Pinette**: Methodology; data curation; formal analysis; writing—original draft. **Nadia Giguère**: Conceptualization; investigation; funding acquisition; project administration; supervision; visualization; writing—review and editing; writing—original draft; methodology. **Matthew Menear**: Validation; writing—review and editing. **Cynthia Cameron**: Validation. **Elyse Marois**: Validation; writing—review and editing. **Brigitte Vachon**: Supervision; conceptualization; investigation; funding acquisition; writing—original draft; writing—review and editing; methodology; project administration; resources.

## CONFLICT OF INTEREST STATEMENT

The authors declare no conflict of interest.

## ETHICS STATEMENT

The study has been carried out with the approval of the ethics committee of the CIUSSS Centre‐Sud‐de‐l'île‐de‐Montréal and the CIUSSS de l'Est‐de‐l'île‐de‐Montréal. All participants have given clear and informed consent via the consent format approved by these committees.

## Data Availability

Some of the data supporting those findings have been implemented into the present articles. The entirety of this data is available from the corresponding author, Justine Labourot, upon reasonable request.
